# Functional phenotyping of the CYP2D6 probe drug codeine in the horse

**DOI:** 10.1186/s12917-021-02788-y

**Published:** 2021-02-13

**Authors:** S. R. Gretler, C. J. Finno, P. H. Kass, H. K. Knych

**Affiliations:** 1 K.L. Maddy Equine Analytical Pharmacology Laboratory, University of California-Davis, School of Veterinary Medicine, 620 West Health Science Drive, Davis, CA 95616 USA; 2 Department of Population Health and Reproduction, School of Veterinary Medicine, University of California, Davis, USA; 3 Department of Veterinary Molecular Biosciences, School of Veterinary Medicine, University of California, Davis, USA

**Keywords:** Codeine, CYP450, Horse, Metabolic ratio, Phenotyping

## Abstract

**Background:**

In humans, the drug metabolizing enzyme CYP2D6 is highly polymorphic resulting in substantial differences in the metabolism of drugs including anti-arrhythmics, neuroleptics, and opioids. The objective of this study was to phenotype a population of 100 horses from five different breeds and assess differences in the metabolic activity of the equine CYP2D6 homolog using codeine as a probe drug. Administration of a probe drug is a common method used for patient phenotyping in human medicine, whereby the ratio of parent drug to metabolite (metabolic ratio, MR) can be used to compare relative enzyme function between individuals. A single oral dose of codeine (0.6 mg/kg) was administered and plasma concentrations of codeine and its metabolites were determined using liquid chromatography mass spectrometry. The MR of codeine O-demethylation [(codeine)/(morphine + morphine-3-glucuronide + morphine-6-glucuronide)] was determined using the area under the plasma concentration-time curve extrapolated from time zero to infinity (AUC_0-∞_) for each analyte and used to group horses into predicted phenotypes (high-, moderate-, and low-MR).

**Results:**

The MR of codeine O-demethylation ranged from 0.002 to 0.147 (median 0.018) among all horses. No significant difference in MR was observed between breeds, age, or sex. Of the 100 horses, 11 were classified as high-MR, 72 moderate-MR, and 17 low-MR. Codeine AUC_0-∞_ and O-demethylation MR were significantly different (*p* < 0.05) between all three groups. The mean ± SD MR was 0.089 ± 0.027, 0.022 ± 0.011, and 0.0095 ± 0.001 for high-, moderate-, and low-MR groups, respectively. The AUC for the morphine metabolites morphine-3-glucuronide and morphine-6-glucuronide were significantly different between high-and low-MR groups (*p* < 0.004 and *p* < 0.006).

**Conclusions:**

The MR calculated from plasma following codeine administration allowed for classification of horses into metabolic phenotypes within a large population. The range of codeine metabolism observed among horses suggests the presence of genetic polymorphisms in *CYP2D82* of which codeine is a known substrate. Additional studies including *CYP2D82* genotyping of high- and low-MR individuals are necessary to determine the presence of *CYP2D* polymorphisms and their functional implications with respect to the metabolism of therapeutics.

## Background

Cytochrome 2D6 (CYP2D6) belongs to the cytochrome P450 (CYP450) superfamily of enzymes, which is involved in the metabolism of drugs and toxicants, as well as the metabolism and synthesis of endogenous substrates such as eicosanoids and cholesterol [1]. While constituting only 2% of total CYP450 content in the liver, CYP2D6 is responsible for metabolizing 25–30% of drugs including antiarrhythmics, antidepressants, anti-cancer drugs, neuroleptics, and opioids [2, 3]. In addition, human CYP2D6 displays high inter-individual variability, with a 1000-fold difference in enzyme activity, largely due to the prevalence of polymorphisms within the CYP2D6 gene [4]. Clinical implications of these polymorphisms are well documented and can result in lack of therapeutic efficacy due to rapid drug metabolism or toxicity and even death in slow metabolizers due to drug bioaccumulation [5–7]. This large variation in CYP2D6 activity has led to the classification of three metabolizer phenotypes; poor (PM), extensive (EM), and ultra-rapid (UM) [8]. Administration of a CYP2D6-selective probe drug and subsequent calculation of the metabolic ratio (MR) of parent drug to metabolite from plasma or urine samples is a common method for the characterization of CYP2D6 phenotypes. Correspondingly, poor, extensive, and ultra-rapid metabolizers are described as having high-, moderate-, and low-MR, respectively. Individuals classified as poor or ultra-rapid metabolizers, based on the MR, are at risk for adverse drug reactions or lack of therapeutic response, respectively when administered CYP2D6 substrates [6, 9, 10].

**Fig. 1:**
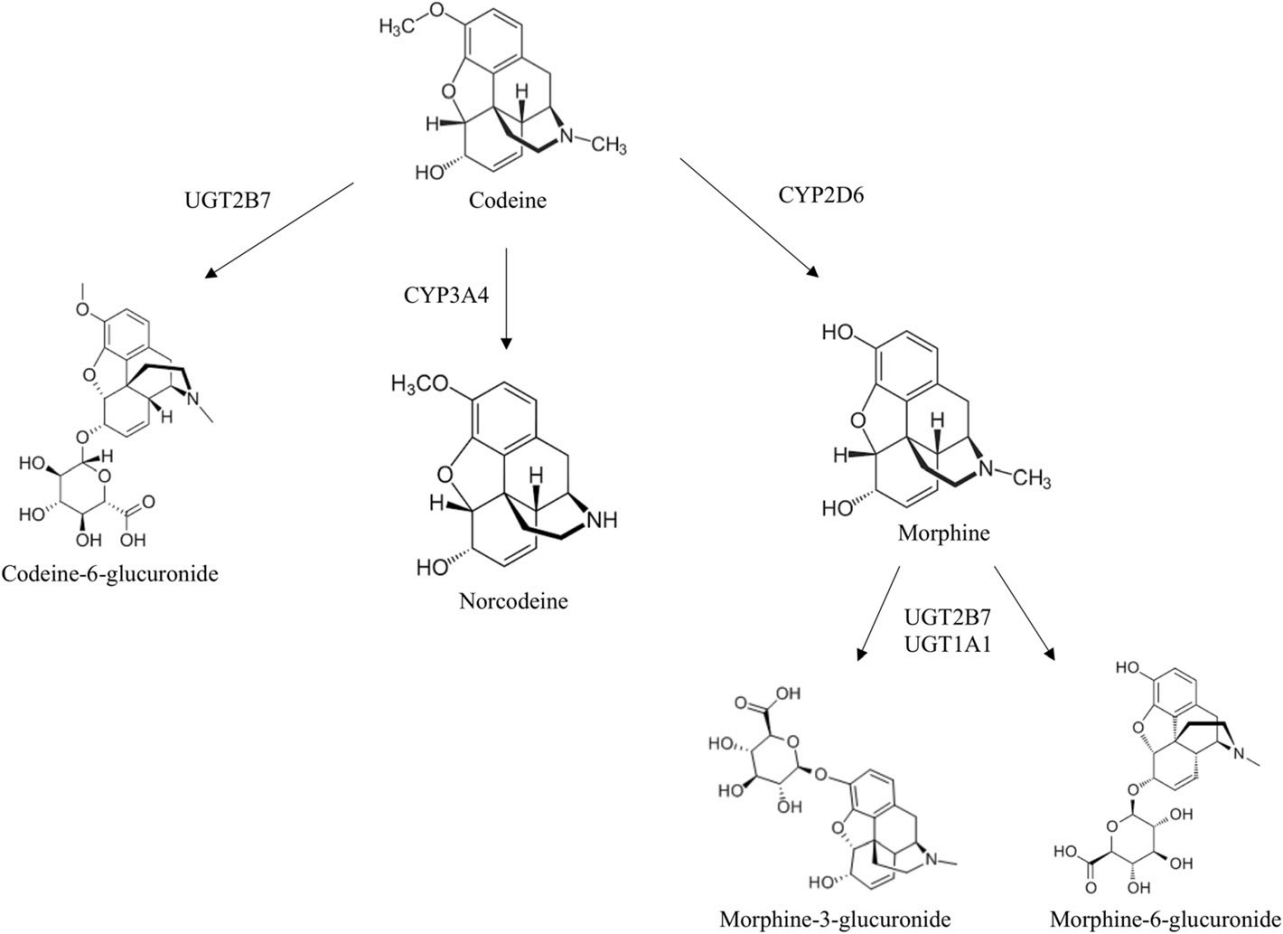
Major codeine metabolites in the human and enzymes responsible for metabolite formation

In the horse, reports of polymorphisms in drug metabolizing enzymes such as CYP450s, and their effect on therapeutic outcomes are sparse. However, previous reports have identified mutations in an equine CYP2D ortholog, CYP2D50, as well as phenotypic differences in the metabolism of CYP2D50 substrates, suggesting a range of enzyme function [11, 12]. While humans possess one active CYP2D gene, other species, such as the marmoset, rat and mouse, have multiple CYP2D genes [13–15]. In the horse, six CYP2D genes have been identified, however, the expression and activity for all has not yet been established [16]. Recently, another CYP2D6 ortholog, CYP2D82 was found to catalyze the O-demethylation of codeine to morphine in vitro [17]. The investigators concluded that codeine was a selective substrate for CYP2D82, as no metabolites were detected in incubations with other expressed equine CYP2D enzymes [17]. Additionally, in vivo studies have demonstrated that codeine is metabolized to the same five main metabolites found in humans, including the CYP2D-generated metabolite morphine (Fig. 1) [18].

While codeine is not commonly used as a probe drug for CYP2D6 in humans, there are numerous reports demonstrating that genetic polymorphisms in CYP2D6 can lead to differing concentrations of the active metabolite morphine between individuals [7, 19]. Differences in morphine concentrations can result in a large degree of individual variability with respect to analgesia, following codeine administration [7, 20]. Furthermore, studies have shown that the MR for codeine O-demethylation in humans is highly correlated with CYP2D6 genotype and that codeine is as effective as the established probe drug, desbrisoquine, in CYP2D6 phenotyping [21, 22]. Given previous evidence of variable CYP2D metabolism in the horse, and the specificity of codeine for the novel equine CYP2D enzyme, CYP2D82, we hypothesized that codeine will be an effective probe drug for the phenotyping of horses. Due to the large number of drugs metabolized by CYP2D6 in the human, if members of the CYP2D family in the horse prove to be as important in the metabolism of therapeutic substances, significant variations in CYP2D enzyme metabolism may result in drug toxicity and lack of efficacy in the horse as is observed in humans [6, 9, 10]. In addition to the clinical implications, decreases in clearance as a result of altered metabolism of CYP2D substrates could lead to positive regulatory findings, despite adherence to established withdrawal times, in performance horses. The goal of the currently reported study is to add to previously published reports describing individual differences in CYP2D metabolic activity between horses.

## Results

Quality control samples prepared in replicates (*n* = 6) for codeine, C6G, norcodeine, morphine, M3G, and M6G were analyzed to determine the intra- and inter-day precision and accuracy of the assay (Table 1). The concentration-response relationship (relationship between calibrators and the LC-MS/MS instrument response) for all analytes was linear (*r* = 0.99). The limit of quantitation for the assay was 0.1 ng/ mL and the limit of detection approximately 0.03 ng/mL for all analytes.

**Table 1:** Intra- and interday accuracy and precision (values for liquid chromatography–tandem mass spectrometry (LC-MS/MS) analysis of codeine and its metabolites, norcodeine, codeine-6-glucuronide (C6G), morphine, morphine-6-glucuronide (M3G), and morphine-6-glucuronide (M6G) in equine plasma

Analyte	Concentration (ng mL^− 1^)	Intraday accuracy (% nominal concentration)	Intraday precision (% relative SD)	Interday accuracy (% nominal concentration)	Interday precision (% relative SD)
**Codeine**	0.75	98.0	4.0	104	4.0
	40	103	2.0	108	2.0
	400	109	0.0	105	1.0
**Norcodeine**	0.75	95.0	4.0	95.0	3.0
	40	95.0	2.0	91.0	1.0
	400	110	2.0	105	1.0
**C6G**	0.75	91.0	4.0	100	3.0
	40	100	1.0	94.0	1.0
	400	96.0	2.0	102	1.0
**Morphine**	0.75	109	5.0	108	5.0
	40	103	2.0	110	2.0
	400	106	2.0	105	2.0
**M3G**	0.75	92.0	4.0	95.0	4.0
	40	109	12.0	107	6.0
	400	110	2.0	108	2.0
**M6G**	0.75	104	5.0	108	7.0
	40	107	3.0	108	2.0
	400	112	2.0	109	1.0

**Fig. 2:**
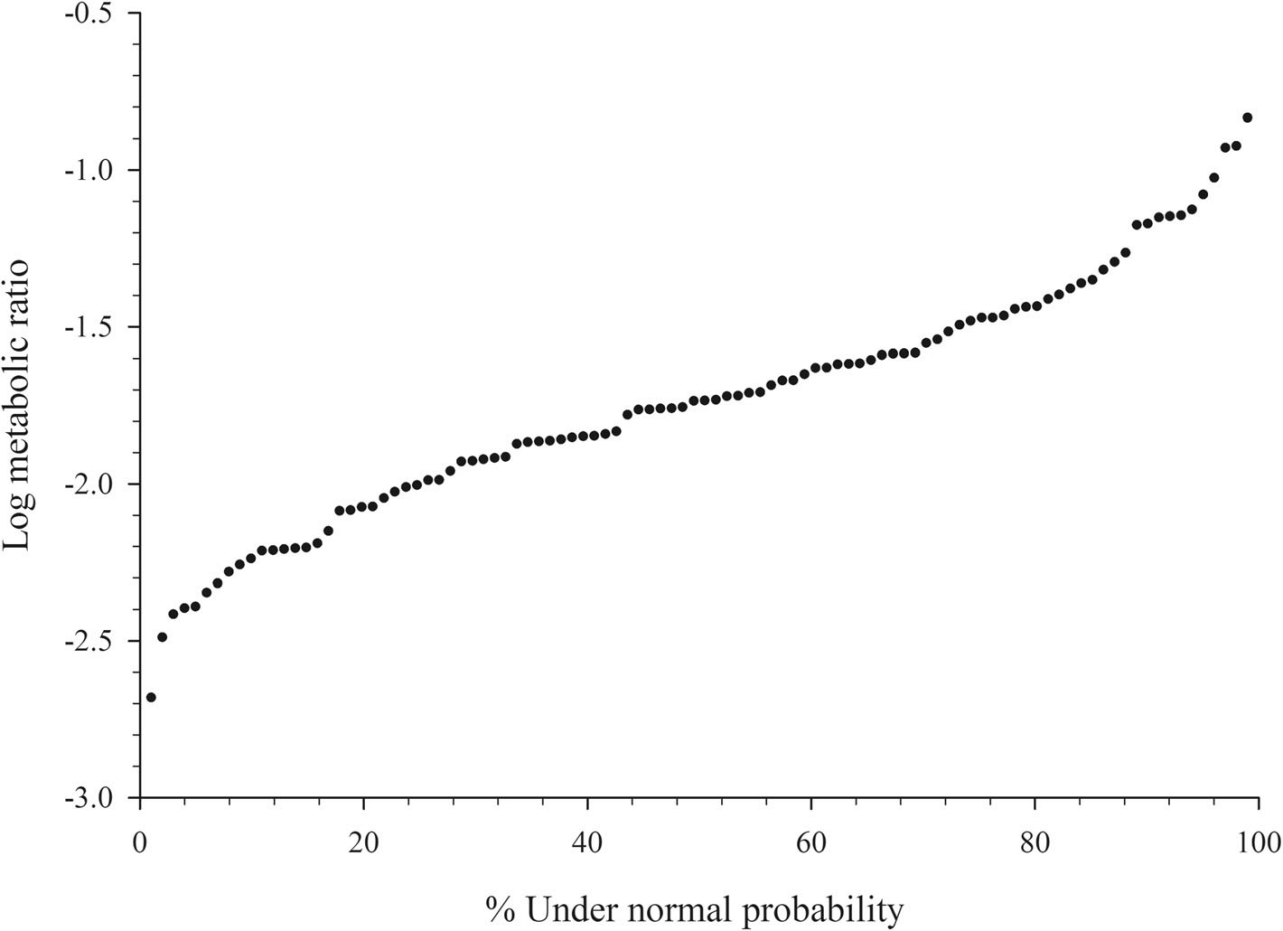
Normal probability plot of logarithmic metabolic ratio [log (AUC_0-∞_ codeine) / (AUC_0-∞_ morphine + AUC_0-∞_ M3G + AUC_0-∞_ M6G)] in 100 horses after a single oral administration of codeine (0.6 mg/kg)

**Fig. 3:**
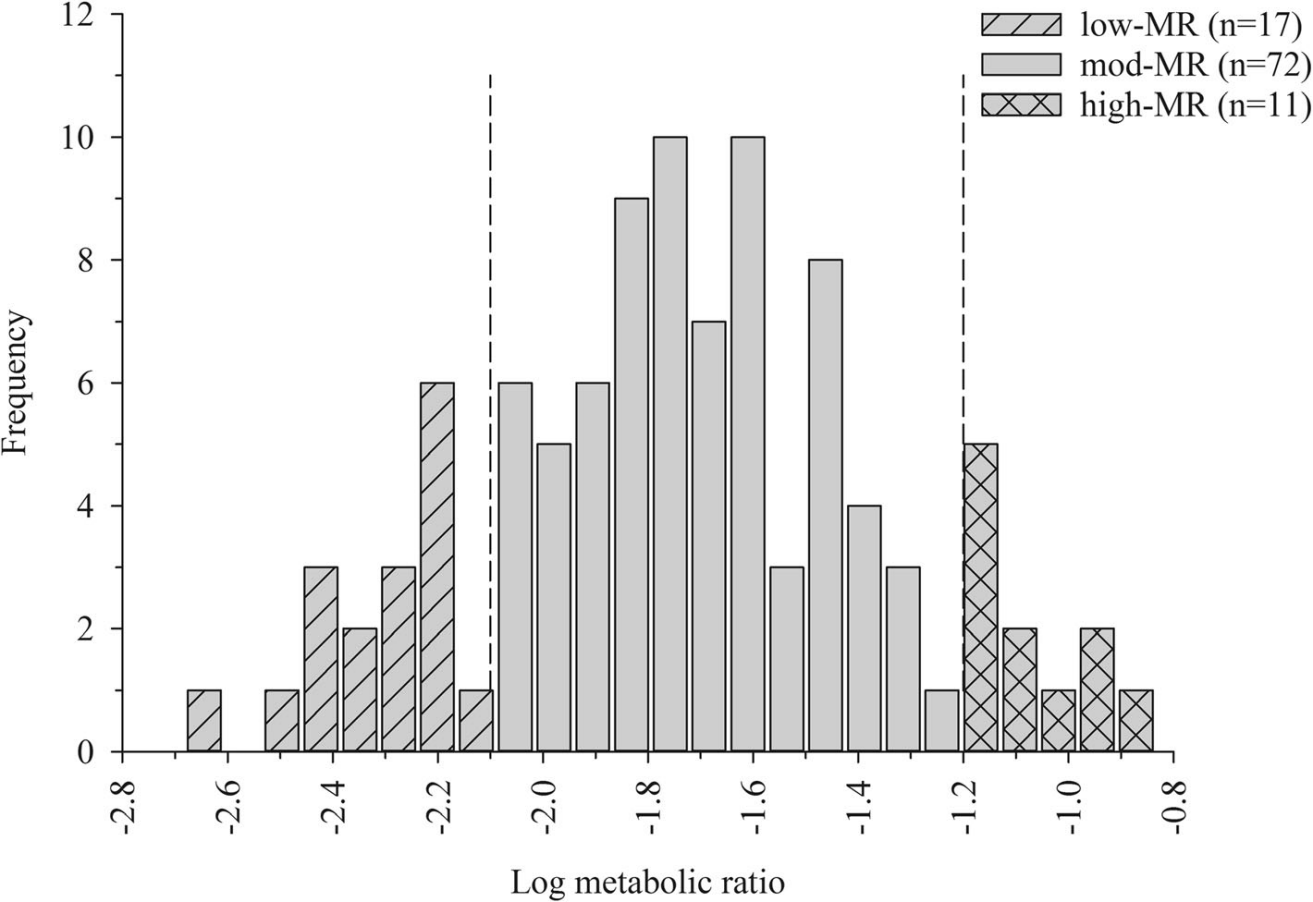
Frequency distribution histogram of log metabolic ratio [log (AUC_0-∞_ codeine) / (AUC_0-∞_ morphine + AUC_0-∞_ M3G + AUC_0-∞_ M6G)] in 100 horses after a single oral administration of codeine (0.6 mg/kg) grouped by predicted phenotype. Dashed vertical lines denote anti-modes

The normal probability plot of log-transformed MRs showed a nonlinear plot indicative of a non-normal distribution [23] (Fig. 2). Visual analysis of the normal probability plot indicated steep break points at both ends corresponding to anti-modes of − 2.1 and − 1.2 using methods previously described [11, 24]. The frequency distribution histogram of log-transformed metabolic ratios confirmed the presence of anti-modes at − 2.1 and − 1.2 by visual analysis (Fig. 3). Anti-modes determined from these plots were used to group horses into predicted phenotypes with 17 classified as low-MR, 11 as high-MR, and the remaining 72 as moderate-MR. The low-MR group consisted of 12 mares and 5 geldings including 11 Thoroughbreds, two Quarter horses, and four warmbloods aged 2 to 20 years. In the high-MR group there were four mares and seven geldings, ten of which were Thoroughbreds and one Standardbred, aged 3–11 years.

The mean (±SD), median, and range of MR for O-demethylation for each breed and age group are presented in Table 2. No significant differences were found for the metabolic ratios between breed, age, or sex. The AUC (mean ± SD and range) for codeine, C6G, morphine, M3G, and M6G, the metabolic ratio, and codeine elimination half-life for the predicted phenotype groups are listed in Table 3 The C_max_ for codeine, C6G, morphine, M3G, and M6G ranged from 4.3–443.1, 18.1–320.9, 0.7–8.3, 113.2–1024.5, and 7.0–87.2 ng/mL, respectively. Metabolic ratios for codeine O-demethylation ranged from 0.002 to 0.147 with a median of 0.018. The AUC for codeine and the MR for O-demethylation were found to be significantly different (*p* < 0.05) between all three predicted phenotypes. Additionally, the AUC for M3G and M6G and the MR for glucuronidation were significantly different (*p* < 0.05) between high- and low-MR groups.

**Table 2:** Metabolic ratio for codeine O-demethylation following administration of a single oral dose to a population of horses of differing breeds and ages. The metabolic ratio calculated as (codeine AUC_0 − ∞_)/(morphine AUC_0 − ∞_ + M3G AUC_0 − ∞_ + M6G AUC_0 − ∞_)

Age (years)	Breed	Number	Metabolic ratio		
			Mean ± SD	Median	Range
0–5	Paint	–	–	–	–
	QH	–	–	–	–
	SB	–	–	–	–
	TB	28	0.029 ± 0.029	0.019	0.002–0.119
	WB	1	0.004 ± 0.00	NA	NA
6–10	Paint	1	0.026 ± 0.00	NA	NA
	QH	2	0.023 ± 0.027	0.023	0.004–0.042
	SB	1	0.023 ± 0.00	NA	NA
	TB	17	0.048 ± 0.038	0.037	0.012–0.147
	WB	5	0.016 ± 0.016	0.010	0.006–0.045
11–15	Paint	–	–	–	–
	QH	7	0.019 ± 0.010	0.017	0.007–0.036
	SB	1	0.072 ± 0.00	NA	NA
	TB	7	0.021 ± 0.022	0.013	0.005–0.067
	WB	4	0.015 ± 0.012	0.011	0.005–0.033
16–20	Paint	–	–	–	–
	QH	2	0.011 ± 0.004	0.011	0.009–0.014
	SB	–	–	–	–
	TB	9	0.012 ± 0.008	0.008	0.003–0.017
	WB	2	0.012 ± 0.003	0.012	0.010–0.014
21–25	Paint	1	0.037 ± 0.00	NA	NA
	QH	2	0.018 ± 0.005	0.018	0.014–0.021
	SB	1	0.029 ± 0.00	NA	NA
	TB	4	0.018 ± 0.007	0.019	0.009–0.024
	WB	2	0.027 ± 0.023	0.027	0.011–0.044
26–30	Paint	–	–	–	–
	QH	1	0.032 ± 0.00	NA	NA
	SB	1	0.014 ± 0.00	NA	NA
	TB	1	0.025 ± 0.00	NA	NA
	WB	–	–	–	–

**Table 3:** Pharmacokinetic values (mean ± SD) for codeine and codeine 6-glucuronide (C6G), morphine, morphine 3-glucuronide (M3G), and morphine 6-glucuronide (M6G) after a single oral administration of codeine (0.6 mg/kg) to horses. Parameters are sorted by predicted phenotypic groups

Parameter	Predicted phenotype		
	Poor (*n* = 11)	Extensive (*n* = 72)	Ultra-rapid (*n* = 17)
Codeine AUC_0-∞_ (h·ng/mL)	257.72 ± 122.93^b,c^	76.49 ± 46.35^a,c^	20.84 ± 6.33^a,b^
C6G AUC_0-∞_ (h·ng/mL)	500.15 ± 249.83	630.12 ± 331.76	523.38 ± 209.18
Morphine AUC_0-∞_ (h·ng/mL)	27.46 ± 9.14	30.82 ± 10.26	34.66 ± 6.31
M3G AUC_0-∞_ (h·ng/mL)	2700.64 ± 892.03^c^	3277.89 ± 935.74	3904.93 ± 987.01^a^
M6G AUC_0-∞_ (h·ng/mL)	98.25 ± 32.76^b,c^	170.32 ± 71.02^a^	180.92 ± 65.70^a^
MR O-demethylation	0.089 ± 0.027^b,c^	0.022 ± 0.011^a,c^	0.0095 ± 0.001^a,b^
MR glucuronidation	0.711 ± 0.596^b,c^	0.153 ± 0.137^a^	0.045 ± 0.020^a^
Codeine *t*_1/2_^d^ (h)	1.32 ± 0.56	2.50 ± 1.37	2.24 ± 0.88

MR for O-demethylation calculated as: (codeine AUC)/(morphine AUC + M3G AUC + M6G AUC)

MR for glucuronidation calculated as: (codeine AUC)/(codeine-6-glucuronide AUC)

*AUC* area under the plasma concentration-time curve, *MR* metabolic ratio, *t*_1/2_ terminal half-life

^a^significantly different than poor metabolizers (*P* < 0.05); ^b^significantly different than extensive metabolizers (*P* < 0.05); ^c^significantly different than ultra-rapid metabolizers (*P* < 0.05)

^d^expressed as a harmonic mean

**Fig. 4:**
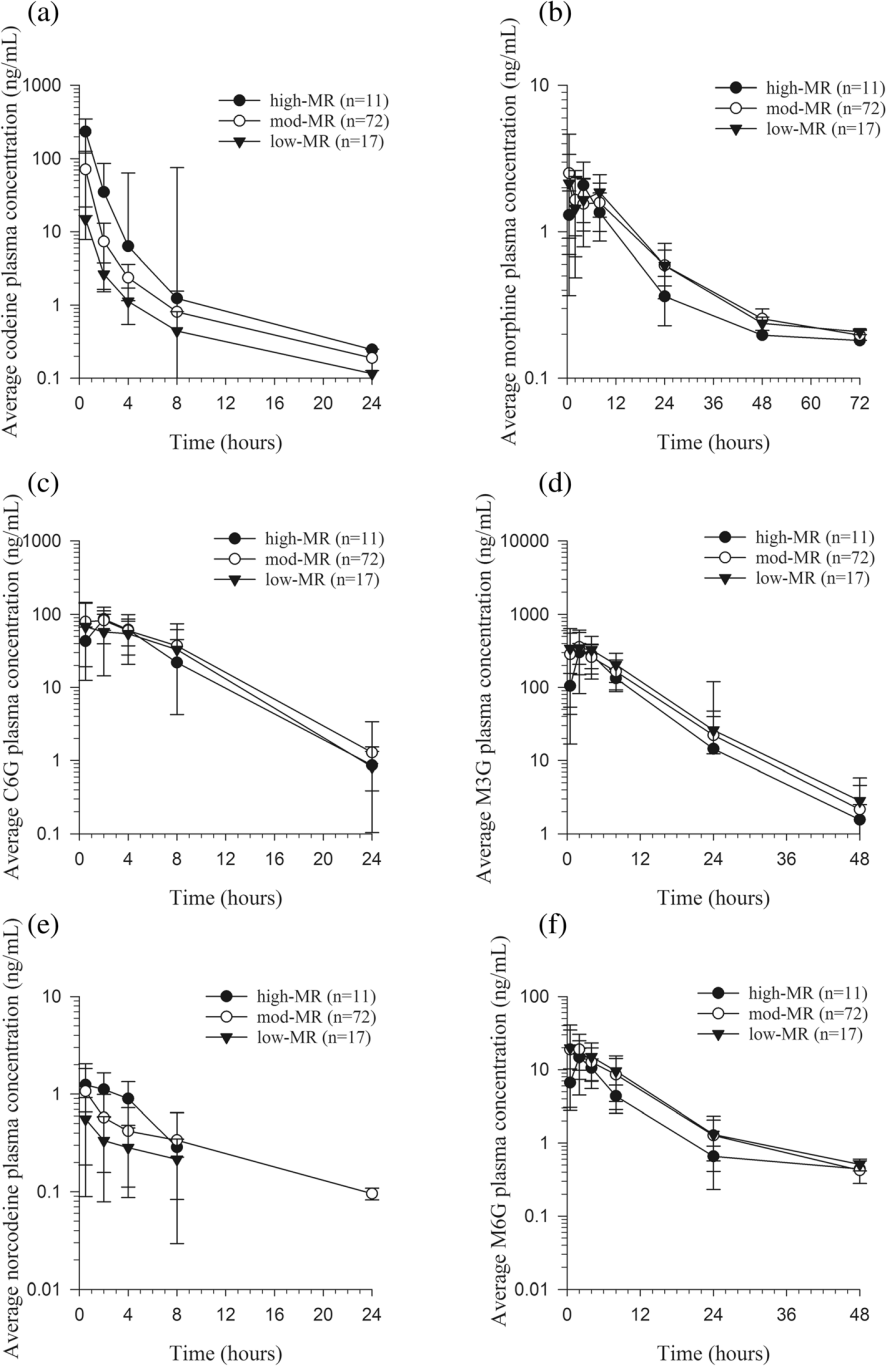
Mean plasma-concentration time curves for codeine (**a**), morphine (**b**), C6G (**c**), M3G (**d**), norcodeine (**e**), and M6G (**f**) based on predicted phenotype in horses following a single oral dose of codeine (0.6 mg/kg)

Average plasma concentrations of codeine and its metabolites were plotted for each phenotypic group (Fig. 4a-f). The average plasma concentrations for codeine and C6G were highest for the high-MR group, followed by the moderate-MR, and lastly the low-MR group. Morphine plasma concentrations were similar for moderate- and low-MR groups, but lower for the high-MR group. Morphine-3-glucuronide plasma concentrations were highest in the low-MR individuals and lowest in the high-MR. The average C_max_ for M6G was 14.84, 25.28, and 25.96 ng/mL for high-, moderate-, and low-MR groups.

## Discussion

Similar to what has been described in humans, there are reports suggestive of varying metabolic capabilities between individual horses [12]. The goal of the current study was to add to the limited knowledge regarding differences in drug metabolism in this species, specifically that mediated by CYP2D82, the equine homolog to the highly polymorphic, CYP2D6. To that end, codeine, which is metabolized to morphine by CYP2D82 [17], was used as a probe drug to screen for differing CYP2D82 metabolic phenotypes in a population of 100 horses. The pharmacokinetics and metabolic profile of codeine has been described previously [18]. In agreement with the previous report, the primary metabolite produced in the current study was the morphine metabolite M3G, followed by C6G, with morphine and M6G present at lower concentrations.

As described previously, the AUC for codeine and its metabolites were determined and subsequently used to calculate a MR (codeine: metabolite) to classify horses into metabolic phenotypes [23]. The codeine t_1/2_ was not significantly different between the predicted metabolic phenotypes and was in accordance with previous reports. Interestingly, the codeine AUC_0-∞_ for moderate-MR individuals was lower than previously reported [18]. While the reason for this is not immediately evident, possible explanations include inter-individual variability within study animals coupled with the smaller sample size in the previous report. Additionally, differences in the pharmacokinetic modeling approach between the studies might account for the observed differences in codeine AUC_0-∞_. The AUC_0-∞_ for codeine was significantly different between predicted metabolic groups (high-, moderate-, and low-MR) with high-MR > moderate-MR > low-MR. Although the AUC_0-∞_ for both C6G and morphine followed the expected pattern based on phenotype groupings (low-MR > moderate-MR > high-MR), this difference was not statistically significant. When evaluating the AUCs from the different metabolic groups, it is important to consider that the AUC can be affected by other factors, notably the extent of absorption and distribution. For this reason, the MR is often considered a better indicator of metabolic capabilities and is used routinely in phenotyping studies. The MR for determination of CYP2D82 activity (O-demethylation) was calculated as the AUC_0-∞_ codeine / (AUC_0-∞_ morphine + AUC_0-∞_ M3G + AUC_0-∞_ M6G) as described previously [6, 25]. Production of M3G and M6G from codeine first requires biotransformation to morphine, therefore the glucuronidated metabolites were included as products of the O-demethylation metabolic pathway in the determination of the MR [25].

The MR frequency distribution in the current study is best described as unimodal, however, the absence of an obvious multi-modal distribution upon inspection of the histogram is not definitive evidence that such a distribution does not exist. Although they can be valuable tools for determination of a non-normal or multi-modal distribution and are commonly utilized in phenotyping studies, the shape of a histogram is dependent on sample size and can therefore be misleading. A normal probability plot is another, often times, more definitive way of assessing normality. In the current study, visual analysis of the normal probability plot of the log MR representing O-demethylation, revealed breakpoints suggestive of three phenotypic groups (high-, moderate-, and low-MR) within the population of horses tested. Furthermore, apparent anti-modes on both sides of the frequency distribution histogram suggest the existence of high- and low-MR groups. In the current analysis of 100 horses, the incidence of predicted CYP2D82 high- and low-MR individuals as classified using the probe drug, codeine, was determined to be 11 and 17%, respectively. Comparably, within the Caucasian population for CYP2D6, 5–10% are PMs (high-MR) and 3–5% are UMs (low-MR) [26]. While, notably, this is the first study of its kind in horses and sample size is limited compared to human studies, results of the current study suggest the existence of differing metabolic phenotypes in horses. Similar to what is observed in humans, there is a high likelihood that differing metabolic capabilities may influence therapeutic responses to CYP2D substrates [27, 28].

In humans, the prevalence of CYP2D6 PMs and UMs can vary significantly among different ethnicities. For example, while 5–10% of the Caucasian population are PMs, only 1% of Chinese are PMs [26, 29]. Similar to the interethnic variation observed in CYP2D6 phenotypes, it is plausible that the prevalence of metabolic phenotypes may vary between horse breeds. The present study evaluated a total of one hundred horses from five different breeds to assess the overall prevalence of polymorphisms within a mixed population. Inter-individual variation in MR was observed within the breeds, while most horses fell within the category of moderate-MR, there were high- and low-MR individuals from several different breeds. While no significant differences were observed in the MR between the breeds, it is important to note that the majority of horses studied were of one breed (Thoroughbreds). This, combined with the presumed relatively low frequency of high- and low-MR individuals, may confound the identification of differences between breeds. Additional studies including larger numbers of horses of other breeds would be needed to determine whether phenotype distribution varies between breeds.

In the current study, the primary focus was on identifying differences in the biotransformation of codeine to morphine that could be attributed to altered CYP2D82 activity. Although it has been well established, based on screening of recombinant equine CYPs, that the biotransformation of codeine to morphine is catalyzed by CYP2D82 [17], it is important to note that glucuronidation of the parent drug (codeine) and N-demethylation to norcodeine also contributes to the overall clearance of this drug in horses [18]. These alternate metabolic pathways may explain the similar codeine terminal half-life between metabolic phenotypes observed in the present study. A decrease in O-demethylation capabilities may be compensated for by the codeine glucuronidation and N-demethylation pathways leading to comparable terminal half-lives between the high- and low-MR groups. Inspection of plasma concentration-time curves for the metabolites supports this theory with slightly higher concentrations of C6G and norcodeine noted in the high-MR group compared to low-MR group.

While the current study theorizes that the higher conversion of codeine to C6G may be a possible compensatory clearance pathway, it should also be noted that studies have identified polymorphisms in genes that code for UGT enzymes in humans [30, 31]. Although the UGT enzyme/s responsible for codeine glucuronidation in the horse have not yet been identified, in humans this reaction is catalyzed by UGT2B7 [32]. The gene coding for UGT2B7 in humans has been shown to be polymorphic and, while there are several studies describing the implications of these polymorphisms on morphine glucuronidation and elimination, there are only a few describing effects on overall codeine clearance (both generation of C6G and morphine glucuronides) and those results appear mixed [33, 34]. Nonetheless, the potential for *UGT2B7* polymorphisms complicates classification of horses into CYP2D82 metabolic phenotypes using codeine as a probe drug. Studies conducted in our laboratory, have shown that similar to humans, morphine is glucuronidated by equine UGT2B7 to M3G (unpublished data). If, as in humans, generation of C6G from codeine is also catalyzed by UGT2B7, and this enzyme is polymorphic in horses, it is plausible that a presumed CYP2D82 high-MR individual may actually be a UGT2B7 UM. In the current study, there does not appear to be a difference in the morphine: M3G MR (calculated as morphine AUC_0-∞_ /M3G AUC_0-∞_), suggesting that the rate-limiting step is production of morphine, which is catalyzed by CYP2D82 and not glucuronidation. Furthermore, the MRs for the codeine glucuronidation pathway (codeine: C6G) are comparable between proposed phenotype groups suggesting that differences between groups are not related to genetic mutations in genes coding for UGT enzymes. However, additional metabolism studies as well as genotyping studies focused on *CYP2D82* and *UGT*s are warranted to definitively prove this theory.

While screening of recombinant equine CYPs have demonstrated that CYP2D82 catalyzes the biotransformation of codeine to morphine, it is important to note that the reported in vitro Km of CYP2D82 was greater than the maximum observed codeine plasma concentrations in the presented report [17]. However, plasma concentrations of codeine may differ significantly from hepatic concentrations, where the majority of CYPs reside. In humans, hepatic concentrations of codeine have been found to be up to 100-times greater than in plasma [35]. Furthermore, differences in in vitro and in vivo conditions may affect enzyme kinetics, such as the less efficient coupling of CYPs with the coenzyme P450 reductase in in vitro experiments. Given this limitation, potential involvement of other phase 1 drug metabolizing enzymes such as flavin-containing monooxygenases (FMOs), or additional unidentified CYPs in the metabolism of codeine in the horse warrants further exploration.

With the reduced cost, and development of rapid DNA sequencing methods, modern phenotyping studies frequently include genotyping to characterize polymorphic alleles within the population and assign activity scores based on in vivo activity [36]. In order to determine a causal link between allele function and metabolic phenotype, genotyping of suspected polymorphic alleles is necessary. However, prior to widespread genetic sequencing and characterization of polymorphic CYP alleles in humans, phenotyping studies used methods of visual analysis of normal probability plots and frequency histograms, as applied in the presented study, to describe proposed metabolic phenotypes [24, 37]. Ongoing studies involving whole genome sequencing are currently underway to determine whether genetic polymorphisms are present in the CYP2D82 gene of horses identified as high- or low-MR in this report. Previous sequencing of the equine CYP2D50 gene identified several exonic SNPs that may affect enzyme activity and metabolism of CYP2D substrates [11, 12]. It is important to note that other factors may influence the metabolism of drugs in vivo, including epigenetic changes, polymorphisms within other genes such as transporters involved in drug absorption [38], age, and environment, such as diet and toxicant exposure [39]. While phenotyping is a valuable tool to assess in vivo metabolism of CYP2D substrates, genotyping horses identified as high- or low-MRs would determine whether observed differences in metabolism arise from altered CYP2D82 activity as opposed to nongenetic or epigenetic differences.

## Conclusions

The administration of codeine, a known substrate of equine CYP2D82, as a probe drug suggests three metabolic phenotypes (high-, moderate-, and low-MR) within a mixed-breed population of horses, as evidenced by significant differences between the MR representing O-demethylation. While the study of polymorphisms in genes coding for metabolic enzymes is very much in its infancy in horses, the potential therapeutic implications, as have been described in human medicine, warrant further study including additional drug metabolism, phenotyping and genotyping studies.

## Methods

### Animals

One hundred horses (2 American Paint Horses, 14 Quarter Horses, 4 Standardbreds, 66 Thoroughbreds, and 14 Warmbloods) were used in this study. Of the 100 horses, 52 were geldings and 48 were mares. Horses ranged in age from 2 to 28 years (11 ± 7) with a mean ± SD weight of 530.6 ± 51.0 kg. The horses used in this study included University-owned research horses and horses from a private Northern California Thoroughbred training facility. The horses were housed in a mixture of pasture, indoor stalls, and stalls with outside runs attached, depending on the facility. The horses were fed twice daily, consisting of AM and PM feedings of grass or alfalfa hay, according to their regular schedule. Prior to participation in the study, all horses were determined healthy by physical examination, complete blood count, and a serum biochemistry panel. The horses did not receive any medications for at least 2 weeks prior to the start of the study. Additionally, no anthelminthics were administered for at least 8 weeks prior to codeine administration. Following the conclusion of the study, all animals were returned to the University research herd or to training. This study was approved by the Institutional Animal Care and Use Committee of the University of California, Davis (#20319).

### Drug administration and instrumentation

Codeine sulfate tablets (30 mg tablets; West-Ward Pharmaceuticals Corp., NJ) were suspended in 6 mL of water in a 20 mL dosing syringe and administered as a single dose of 0.6 mg/kg into the oral cavity. Manual shaking of the dosing syringe was performed to ensure adequate suspension prior to administration. The dosing syringe was rinsed with an additional 6 mL of water and delivered to the oral cavity immediately after dosing. Horses were not fasted prior to drug administration and were weighed immediately prior to drug administration.

### Sample collection

Blood samples were collected by direct jugular venipuncture into ethylenediaminetetraacetic acid (EDTA) containing blood tubes (Covidien, MA, USA) immediately before drug administration (time 0) and at 0.5, 2, 4, 8, 24, 48, and 72 h post administration. Samples were placed on ice until centrifugation at 3000 ***g*** for 10 min. Plasma was transferred to cryovials (Phenix Research Products, Chandler, NC, USA) and stored at − 20 °C until analysis.

### Codeine plasma concentration determination

Quantitative analysis of plasma samples was performed using liquid chromatography tandem mass spectrometry (LC-MS/MS). Plasma samples, calibrators, and negative controls were all prepared as previously described [18]. The previously published method was adapted for the TSQ Altis triple quadrupole mass spectrometer coupled with a Vanquish liquid chromatography system (Thermo Scientific, San Jose, CA).

The analyte concentrations were measured in equine plasma by LC-MS/MS using positive heated electrospray ionization (HESI(+)). Quantitative analysis was done on a TSQ Altis triple quadrupole mass spectrometer with a Vanquish liquid chromatography system (Thermo Scientific, San Jose, CA). The parameters were as follows; spray voltage 3500 V, vaporizer temperature 350 °C, sheath gas 50 (arbitrary units), and auxillary gas 10 (arbitrary units). Product masses and collision energies of each analyte were optimized for the TSQ Altis by infusion of the standards into the machine. Chromatography was performed using a Zorbax Eclipse-XDB-Phenyl 3x100mm, 3 μm column (Agilent Technologies) and a linear gradient of ACN in water with 0.2% formic acid and a 0.45 ml/min flow rate. The ACN concentration was held at 5% for 0.5 min initially, then gradually increased to 30% over 4.0 min, then to 90% over 0.01 min, kept at 90% for 0.2 min, and returned to 5% for 2.3 min.

Selective Reaction Monitoring (SRM) was used to detect and quantify the initial precursor ion for codeine (mass to charge ratio 300.1 *(m/z)*), C6G (476.1 *(m/z)*), norcodeine (286.1 *(m/z)*), morphine (286.1 *(m/z)*), M3G (462.2 *(m/z)*), M6G (462.1 *(m/z)*), and the internal standards d6-codeine (306.1 *(m/z)*), d6-morphine (292.1 *(m/z)*), and d3-M6G (465.2 *(m/z)*). Responses for the following product ions were plotted and Quanbrowser software (Thermo Scientific) was used to integrate the peaks at the corresponding retention time: codeine (*m/z* 165, 215, 199), C6G (*m/z* 300, 215, 165), norcodeine (*m/z* 165, 201), morphine (*m/z* 165, 201), M3G (*m/z* 286), M6G (*m/z* 286) and the internal standards d6-codeine (*m/z* 165, 218), d6-morphine (*m/z* 201), and d3-M6G (*m/z* 289). Linear regression using Quanbrowser software was employed to create the calibration curves and quantitate the analytes using a 1/X weighting factor.

### Pharmacokinetic analysis

Noncompartmental analysis of plasma codeine, C6G, morphine, M3G and M6G concentrations were performed using Phoenix WinNonlin Version 8.2 (Certara, Princeton, NJ, USA). The analysis included plasma concentrations above the limit of quantitation (LOQ) for the respective analytes. Elimination half-life (*t*_1/2_) was calculated with the formula *t*_1/2_ = (ln2)/λ_*z*_, where λ_*z*_ is the elimination rate constant determined as the terminal slope of the plasma concentration-time curve. Area under the curve from time 0 to infinity (AUC_0-∞_) was determined using the log up-linear down trapezoidal method extrapolated to infinity by dividing the last measured plasma concentration by the terminal slope.

Metabolic ratios (MR) were calculated using the following formulas:

$$ \mathrm{MR}\ \mathrm{codeine}\ \mathrm{O}-\mathrm{demethylation}=\left({\mathrm{AUC}}_{0-\infty }\ \mathrm{codeine}\right)/\left({\mathrm{AUC}}_{0-\infty }\ \mathrm{morphine}+{\mathrm{AUC}}_{0-\infty }\ \mathrm{M}3\mathrm{G}+{\mathrm{AUC}}_{0-\infty }\ \mathrm{M}6\mathrm{G}\right) $$

$$ \mathrm{MR}\ \mathrm{codeine}\ \mathrm{glucuronidation}=\left({\mathrm{AUC}}_{0-\infty }\ \mathrm{codeine}\right)/\left({\mathrm{AUC}}_{0-\infty }\ \mathrm{codeine}-6-\mathrm{glucuronide}\right) $$

The Kolmogorov-Smirnov test was used to assess the normality of the MR data. Metabolic ratios were logarithmically transformed and plotted as a frequency histogram. Probit transformations of the data were made by plotting log MR against the corresponding percent area under the probability curve on probability paper. Percent area under the probability curve was calculated as 100 x *i* / (n + 1) where *i* is the rank of the MR for each horse from lowest to highest value and n is the number of samples. Visual inspection of the graphs for breakpoints indicated anti-modes separating phenotypes [24].

Statistical analyses were conducted using commercially available software (Stata/IC 15.1, StataCorp LP, TX). Statistical comparisons of mean differences between the AUC_0-∞_, MRs, and *t*_1/2_ of the phenotypic groups were performed using a one-way analysis of variance (ANOVA). If the overall group effect was significant (*p* < 0.05), then pairwise comparisons were performed using Scheffe’s method for multiple comparison adjustment.

## Data Availability

The datasets used and/or analyzed during the current study are available from the corresponding author on reasonable request.

## Abbreviations

AUC: Area under the curve
CYP450: Cytochrome P450
C6G: Codeine-6β-D-glucuronide
EM: Extensive metabolizer
LC-MS/MS: Liquid chromatography mass spectrometry/mass spectrometry
LOQ: Limit of quantitation
M3G: Morphine-3β-D-glucuronide
M6G: Morphine-6β-D-glucuronide
MR: Metabolic ratio
PM: Poor metabolizer
UM: Ultra-rapid metabolizer
